# Overview on the Effects of *N*-Acetylcysteine in Neurodegenerative Diseases

**DOI:** 10.3390/molecules23123305

**Published:** 2018-12-13

**Authors:** Giuseppe Tardiolo, Placido Bramanti, Emanuela Mazzon

**Affiliations:** IRCCS Centro Neurolesi “Bonino-Pulejo”, Via Provinciale Palermo, Contrada Casazza, 98124 Messina, Italy; gstardiolo@gmail.com (G.T.); bramanti.dino@gmail.com (P.B.)

**Keywords:** *N*-acetylcysteine, glutathione precursor, oxidative stress, Parkinson’s disease, Alzheimer’s disease, neuropathic pain, stroke

## Abstract

*N*-acetylcysteine (NAC), which is an acetylated cysteine compound, has aroused scientific interest for decades due to its important medical applications. It also represents a nutritional supplement in the human diet. NAC is a glutathione precursor and shows antioxidant and anti-inflammatory activities. In addition to the uses quoted in the literature, NAC may be considered helpful in therapies to counteract neurodegenerative and mental health diseases. Furthermore, this compound has been evaluated for its neuroprotective potential in the prevention of cognitive aging dementia. NAC is inexpensive, commercially available and no relevant side effects were observed after its administration. The purpose of this paper is to give an overview on the effects and applications of NAC in Parkinson’s and Alzheimer’s disorders and in neuropathic pain and stroke.

## 1. Introduction

**Figure 1:**
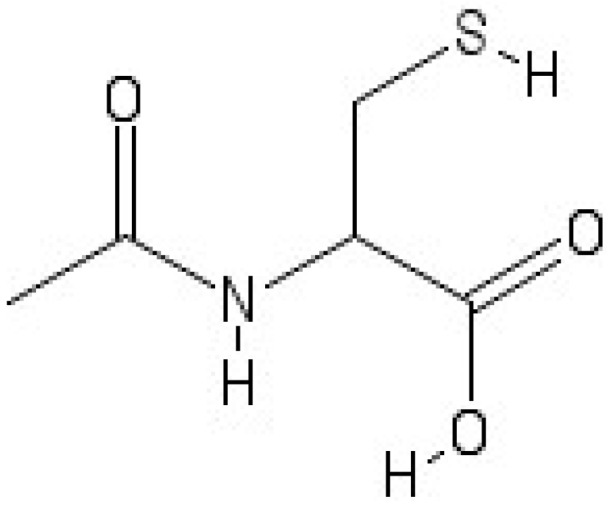
Structure of the *N*-acetylcysteine (NAC).

*N*-acetylcysteine (NAC) (Figure 1) is a precursor of l-cysteine and it is widely used in mucolytic therapy and used to treat paracetamol overdose. NAC is recognized by the World Health Organization (WHO) as a relevant medication required in a basic health system. NAC may be administered orally by inhalation or intravenously and it is also listed as an essential medicine [1]. Moreover, NAC is considered a medication that displays pro-neurogenic and neuroprotective properties. It is also used in therapies to counteract neurodegenerative and psychiatric diseases [2,3]. As reported in the literature, the single use of glutathione (GSH) as oral medication does not sufficiently recover GSH levels. In fact, in body districts such as liver and intestines, GSH is quickly hydrolyzed [4] and its capacity to cross through the blood-brain barrier (BBB) is insufficient. In the same way, the oral intake of l-cysteine has also shown poor effects on the recovery of GSH in the brain due to its metabolic activity [5,6,7]. An entirely different issue is suggested by oral NAC administration. Its use results in the increase of plasma cysteine levels that finally involve a subsequent increase in plasma GSH [8,9]. Studies on animal models [10,11], cited in scientific literature, have described that NAC shows the activity to efficaciously penetrate the BBB increasing the GSH levels in the brain. This peculiarity of NAC may be a crucial factor in neurological disorders therapies in which variations in GSH levels and redox pathways were observed. Likewise, several forms of neuronal damage and degeneration are associated with the excitotoxic damage that it is related to the activation and regulation of *N*-methyl-d-aspartate (NMDA) glutamate receptors. Among its various properties, NAC has shown antioxidant and anti-inflammatory properties. As an antioxidant, NAC neutralizes free radicals before they may cause damages in cells. NAC increases the levels of cysteine/GSH in cells and acts as a scavenger of oxidant species. Representing an acetylated cysteine compound with an acetyl group bonded to the nitrogen atom, NAC may be oxidized by a variety of radical compounds [12]. The actions of NAC consist of restoring the antioxidant potential in cells by replenishing the depletion of GSH by free radicals and in scavenging the reactive oxygen species (ROS). As an anti-inflammatory substance, NAC is able to limit the release of cytokines in the early state of immune proliferation [13].

Thanks to these previously mentioned various activities, there is a growing interest in investigating the favorable effects of NAC in neurodegenerative diseases. In this paper, our attention is focused on the potential effects and applications of NAC in Parkinson’s disease and Alzheimer’s disease in neuropathic pain and stroke.

## 2. NAC as Antioxidant and Anti-Inflammatory Compound

In several cellular systems, NAC promotes effects aimed to maintain the survival functions of cells, which also induces the production of intracellular GSH known as the principal antioxidant produced by the body that protects cells from oxidative stress and maintains the redox state inside them [14]. The antioxidant activity of NAC may be associated with rapid interactions with radicals such as ^•^OH, ^•^NO_2_, CO_3_^•−^ and, in the restoration of damaged targets, in essential cellular elements. NAC’s interactions with superoxide (O_2_^•−^), hydrogen-peroxide (H_2_O_2_), and peroxynitrite (ONOO^−^) are relatively slow. This causes questions about the relevance of such reactions in physiological conditions. The peculiarity of NAC may be related to the reduction of disulphide bonds into the proteins, which changes the structure and inhibits the bond with their ligands. Furthermore, bigger reducing compounds are competitors of NAC into sterically less accessible spaces and in GSH synthesis. Reactive species such as ROS play a role in the oxidation of lipids, proteins, and DNA and induce cell injuries that may cause cell death. These activities are involved with the onset of neurodegenerative diseases. The scavenging of ROS and RNS (Reactive Nitrogen Species) together with the bond with redox-active metal ions that are implicated in the catalysis of ROS and RNS formation and the increase of endogenous antioxidant defenses are included in the endogenous antioxidant defense mechanisms. In addition, the use of exogenous antioxidants might be able to reduce damages caused by oxidative stress. Due to this, it is possible to argue if NAC can effectively function as a good antioxidant. It is known that NAC does not react with O_2_ and nitric oxide (NO). It was reported that the rate constant of NAC in the interactions with H_2_O_2_, O_2_^•−^ and ONOO^–^ result is poor. For this reason, their relevance under physiological conditions is doubtful [12]. On the contrary, as mentioned beforehand, NAC reacts with highly oxidizing radicals and may create bonds with redox-active metal ions. Additionally, the thiols can also provide radio-protection by donating reducing equivalents. An example of this type of reaction, which is also called repair reaction, is represented by the carbon-centered radicals that are generated on the structure of DNA or proteins by ^•^OH attack that may be restituted by hydrogen donation from RSH (notation used for a compound also known as Thiol). This kind of process is probably efficacious in conditions of hypoxia in which thiols are in competition with O_2_ for the carbon-centered radicals. GSH is not a relevant radio protector inside cells [15] and other types of thiols or reducing systems may be involved to counteract radiation [16]. Moreover, the protection against cell killing induced by ionizing radiation is not performed by NAC [12]. Cysteine is mainly transported in the alanine-serine-cysteine (ASC) system that represents a ubiquitous system of Na^+^-dependent neutral amino acid transportation [17,18]. Nevertheless, NAC delivers cysteine in cells in an exclusive form not requiring an active transport due to being a membrane-permeable cysteine precursor [19]. NAC, in its free form, after its entrance in cells, is quickly hydrolyzed to permit the release of cysteine, which represents a GSH precursor. GSH, in turn, through the co-actions of γ-glutamylcysteine synthetase (γ-ECS) and GSH synthetase (GSS) is finally synthesized. The γ-ECS may be inhibited by feedback from GSH. GSH synthesis depend on the substrates available in which cysteine commonly represents a limiting precursor [20,21]. Additionally, GSH reductase (GSR) requires NADPH for its functioning to hold intracellular GSH in its thiol form [19]. In order to exert its protective effect to counteract oxidative stress generated by ROS, GSH participates with the reactions both in a non-enzymatically and enzymatically way while the degradation of H_2_O_2_ and hydroperoxides is catalyzed by GSH peroxidase (GPx) [20]. Therefore, an important element that protects cells from oxidative damage is intracellular GSH and the increase of its levels is influenced by NAC [14]. With regards to anti-inflammatory activity, several research studies have observed that NAC is able to limit cytokines release in the early state of immune proliferation [13]. Furthermore, factors such as the tumor necrosis factor alpha (TNF-α) and interleukins as IL-6 and IL-1β may be reduced by NAC in humans with hemodialysis or septic shock [22,23]. Additionally, in animal models, inflammatory markers induced by lipopolysaccharides are influenced by the modulation of NAC [24]. The NF-κB activity is targeted to control the inflammation cascade and the bonding between NF-κB and I-κB prevents its nuclear translocation. The separation of I-κB through phosphorylation operated by IKKβ permits its degradation by the proteasome with a subsequent transportation of NF-κB into the nucleus. The reduction of NF-κB activity is related to the anti-inflammatory effect of NAC [25]. Nevertheless, the activity of IKK is also inhibited by NAC [26]. In effect, the activity and the expression of such transcription factors may be regulated by NAC [12]. This aspect can lead us to consider that the production of some proteins that inhibit the activation of IKKβ/NF-κB axis is supported by NAC.

## 3. Effects of NAC on Neurotransmission

NAC can modulate several key neurotransmitter systems such as glutamate, which are studied because it is involved in a range of mental illness [27,28]. Several neuropsychiatric sicknesses such as schizophrenia [3,29] and addiction [3,30] are related to the dysfunction of the glutamate system [31]. For this reason, physiological activities that regard the regulation of glutamate production, release, concentration in the synapses, and its recycling are strictly under control. Several forms of neuronal damage and degeneration are associated with the excitotoxic damage that is caused by the hyper activation of the NMDARs [32]. To operate their activation, the binding of glutamate and the postsynaptic depolarization are required. When they are activated, Ca^2+^ entry is mediated by NMDARs [33]. The onset of various neurological and psychiatric conditions is related to NMDARs dysfunction in which alterations in receptor-channel function, subunit expression, trafficking, or localization can occur. The hyperactivity or the hypofunction of NMDARs may cause deleterious events, which represents the origin of various nervous system diseases connected to or even generated by the dysfunction of synapses [34]. Central nervous system (CNS) diseases may be affected by altered NMDARs presence or functions in which an excessive activation might lead to neuronal death. For instance, in the case of stroke [35] or as observed in schizophrenia [36], a reduced activity might cause an alteration of the balance between excitation and inhibition in neural circuitry to influence the functions of the CNS. The NMDA receptors are located in glutamatergic synapses, which are present on excitatory and inhibitory neurons. The structure of the subunit or the sub-synaptic location of NMDARs may also influence their contribution in CNS diseases at excitatory synapses [37]. The major excitant neurotransmitter in the mammalian brain is represented by glutamate in which, through ionotropic and metabotropic glutamate receptors (iGluRs and mGluRs), it is implicated in excitatory postsynaptic transmission. Three classes of glutamate-gated channels are known and a group of G-proteins associated with glutamate receptors induce the mobilization of Ca^2+^ from internal reserves [38,39]. 

Receptors such as the α-amino-3-hydroxy-5-methyl-4-isoxazole-propionic acid (AMPA) activated receptors and the NMDARs show a relevance in the long-term adaptive processes [40]. A fundamental component for the control of extracellular glutamate and feedback regulation of glutamate release is represented by the cystine/glutamate antiporter, which is also stated as an x(c)-system or System xc- or Sxc- [41]. This transporter is mainly present in the astrocytes and carries out an action that results to be the fundamental determinant of the non-vesicular release of glutamate. It has been observed that mGluR2/3 present on presynaptic neurons are activated by extracellular glutamate. Moreover, extracellular glutamate regulates vesicular glutamate neurotransmission [41]. Glutamatergic neurotransmission modulation may be influenced by NAC intake, which represents a compound that may induce the activation of the x(c)-system by providing supplementary cystine. In studies that concern psychotomimetic-induced models of schizophrenia, NAC shows beneficial effects through the regulation of the previously mentioned system. Its activity has improved behavioral deficits and inverted the increase of extracellular glutamate [42]. Furthermore, other beneficial effects of NAC have been noted in animal models of cocaine dependence and was indicated to be stimulated by the x(c)-system and presynaptic mGluR2/3 signaling [29,43]. Additionally, for the regulation of the release of glutamate, one more relevant mechanism carried out by NAC consists of modulating the activities of NMDARs [44,45]. Studies have been targeted on the modulation of redox state by GSH. This is due to the fact that sub-toxic levels of oxidant species can regulate NMDAR. In fact, high levels of oxidizing agents generate an effect that reduces the NMDAR activity through the bond with the extracellular redox-sensitive sites and, as a consequence of this, the reduction of GSH has recapitulated such condition [46]. Taking into consideration what has been examined on this specific mechanism of action, the results that were drawn by scientific literature indicate that NAC may regulate directly and indirectly glutamatergic neurotransmission. This activity of NAC may be attributed to its curative action, considering the crucial role of glutamate signaling in neuropsychiatric diseases [47,48].

## 4. NAC in Parkinson’s Disease 

Parkinson’s disease (PD) represents a neurodegenerative disease that interests the CNS and its onset is related to the idiopathic degeneration of cells involved in dopamine production [49]. Symptoms generally show a slow progression and this aspect is different from one person to another due to the diversity of the disorder. Classical clinical signs observed in this particularly neuro-degeneration consist of rigidity on passive movements, tremor at rest, bradykinesia, and hypokinesia. Among the mentioned ailments, some aspects regularly develop with the progression of the disease such as postural instability, orthostatic hypotension, and dementia. Currently, symptoms can be relieved even though there is no definitive cure for PD. Efficient treatments use l-dihydroxyphenylalanine (l-dopa), which is also named Levodopa, to replace dopamine (DA) loss. Levodopa represents a preeminent pharmacological treatment for PD even though the development of motor fluctuations and drug-induced dyskinesia limit its application [50,51]. Despite this, since l-dopa has been introduced in clinical treatment, at some stage of disease progression, it is noted that survival with PD has been considerably prolonged [52]. Furthermore, dopamine agonists are also considered in PD treatment and whether it is combined to l-dopa or not because their action is involved on DA receptors and mimicks the same functions of endogenous DA. Nevertheless, the application of l-dopa is necessary to control the symptoms in patients affected by this disorder. Multi-factorial aspects such as genetic and environmental factors work on the genotype and seem to be related with PD onset [53]. The aging represents an important risk factor undoubtedly involved in the progression of PD. In the brain, a reduction in antioxidant activity and damage of mitochondrial bioenergetics are events related to aging [54,55]. In the substantia nigra of patients that show an initial phase of PD, a relevant reduction of reduced GSH has been observed. This may cause morphological mitochondrial damage through the action of ROS [56]. The first sign recognized of the substantia nigra degeneration is the reduction of GSH and this deficiency seems to travel at the same level of the severity of the illness itself. An increase in ROS and RNS formation seems to be related to GSH reduction. This condition leads to the inhibition of Complex I with the following alteration of the mitochondria that results in a reduction of the GSH production that causes dopaminergic cell death [57]. Moreover, the accumulation of toxic species of α-synuclein plays a crucial role in PD. Such toxic species are increased by oxidative stress and through oxidative ligation to dopamine [58,59]. This indicates that the increase of oxidative stress may lead to a major toxicity of α-synuclein in dopaminergic neurons. Taking into consideration the oxidative damage, some antioxidant compounds have been used in clinical studies for PD treatment [60,61]. Studies that were performed on the involvement of GSH reduction in PD may have pathways aimed at maintaining or restoring the levels of GSH. Additionally, the application of GSH as medication shows some limits because high doses are required to reach therapeutic effective levels, which shows a short half-life in human plasma that reaches less than 3 min after administration and an insufficient capability to penetrate cell membranes [62]. A limiting factor implicated in the GSH synthesis rate in physiological conditions is the cellular availability of cysteine despite that, at high concentrations, cysteine is toxic as a consequence of free radicals generation during its autoxidation [63]. For this reason, in order to increase neuronal GSH levels, substances that can be metabolized to cysteine or act as its precursor have been taken into consideration. Thanks to its chemical properties, NAC may have a relevant part in PD treatment providing cysteine to the brain in which it plays an action as a precursor for the production of GSH and in the stimulation of cytosolic enzymes involved in GSH regeneration [64,65]. Studies have shown that NAC shows properties in crossing the BBB, in the protection of brain mitochondria, and in counteracting the memory loss because of age. This has been observed by analyzing increased activities of Complex I in mitochondria of pre-synaptic terminals of aged mice [54,64]. The contribution of NAC to GSH repletion may represent an important aspect, which makes such a compound optimal against damages in mitochondria in the substantia nigra considering that GSH levels can be reduced as the disease progresses [66]. Additionally, studies carried out on cultured human cortical neurons [67] have highlighted that NAC may protect against the programmed cell death (PCD) induced by DA and in synaptic mitochondrial preparations in aged mice. It has been observed that NAC may enhance the specific activity of complex IV in mitochondria [54,65]. Therefore, it should be important to consider that, under NAC administration, the following benefits have been observed: increase in levels of GSH in the brain of mice [65,68,69], oxidative damage reduction [66], increased brain synaptic and non-synaptic connections [65,70], and the expression of mitochondrial Complex I, which reduces cell death induced by DA [67,71]. Taking into account what has just been stated and highlighted in scientific literature, the inhibition of the Complex I, caused by the extended reduction of GSH, may be caused partly to a reversible event, correlated with age, that involves residues of cysteine with a progressive effect on its enzymatic activity. If GSH is restored to normal levels, the process can be reversible, which indicates that therapeutics, aimed at the preservation of the amount of GSH in dopaminergic neurons, could be helpful. NAC can re-establish the loss in Complex I activity, which shows a role on cysteine residues just as observed among in vitro studies [65,72]. In a mouse model, under oral intake, NAC has shown protective effects against the damage in dopaminergic terminals connected to over-expression of α-synuclein. This indicated that the treatment with NAC in α-synuclein over-expressing mice increased the striatal tyrosine hydroxylase positive terminal density whereas no increase was observed in α-synuclein over-expressing mice with a controlled diet. The reduction in α-synuclein immuno-labelling in the brains of over-expressing mice that have received NAC treatment is also associated with this aspect. In addition, the levels of GSH in the substantia nigra of transgenic mice over-expressing α-synuclein considerably increased with NAC integration [73]. A potential activity performed to counteract PCD in post-mitotic cells and oligodendrocytes by NAC has been highlighted in in vitro experiments [67]. It is plausible that NAC may act in vivo preventing the ROS increase and counteracting PCD since variations in the structure and function of mitochondria are initial phases in apoptosis [74]. In the brain of mice, NF-κB signaling has been influenced by NAC during a long-term treatment, which shows an increase of the cytoplasmic retention of NF-κB that has prevented its action as a transcription factor in the nucleus [73]. In PD models, a further helpful action of NAC has been observed in which a counteracting alteration of sulfhydryl residues in proteins is implicated in regulating cell endurance and in the NF-κB path associated with a reduced activity of NF-κB in such models because an increase in the activation of NF-κB may contribute to disease onset [75,76]. Another particular aspect of NAC consists of its effect on the inhibition of PCD induced by TNF-α in human neuronal cells through the protection of the integrity and function of mitochondria. This aspect is due to NAC ability to prevent in part the membrane depolarization generated by such a cytokine [67,77]. In addition, a further application of NAC is related to a study where this compound has shown to exert an activity as a scavenger of H_2_O_2_ and toxic quinones generated by DA, which has also protected the inhibition of Na^+^, K^+^-ATPase activity mediated by DA. This indicates an additional mechanism of NAC that encourages its use in PD treatment [78]. Therefore, acting against Na^+^, K^+^-ATPase inhibition, NAC may neutralize the death of dopaminergic neurons, which counteracts intracellular damage pathways.

## 5. NAC in Alzheimer’s Disease

Alzheimer’s disease (AD) represents a destructive neurodegenerative disease correlated with age and, currently, there is no definitive treatment. This devastating pathology shows symptoms characterized by the gradual loss of memory leading to dementia. Daily activities are compromised and are affected by cognitive decline that damages behavior, speech, and visual-spatial perception. AD cases appear mainly in elderly people with an impact of 60–70% [79] and some risk factors may include genetic, epigenetic, dietary, and lifestyle [80]. Among them, aging represents the principal risk factor. Therapeutic approaches on AD pathological hallmarks are focused on the idea that the increase of some proteins as amyloid β-peptide (Aβ) produces neuronal damage and death. It has also been observed that reactive species may contribute to AD initiation and progression determining cellular oxidative damage [81]. Specific, cognitive impairment is related to events represented by the death of cholinergic neurons located in the basal forebrain section [82]. A shortfall in acetylcholine (ACh) has been noticed in the AD brain as well as in cholinergic markers as acetylcholinesterase (AChE) and choline acetyltransferase (ChAT) [80,83]. AD physiopathology is commonly linked with amyloid cascade, which shows an increase of the insoluble amyloid protein that leads to the formation of extracellular neuritic amyloid plaques [84]. Further pathological aspects highlighted in AD comprehend: Neuropil threads, found in the post-mortem AD brain [85], inflammatory alterations with astrocytosis and microgliosis [86], intracellular neurofibrillary tangles (NFTs) composed of the misfolded microtubule-associated tau protein, abnormally phosphorylated, which forms part of the cytoskeleton of neurons [87], oxidative stress [81,88], and additional neurochemical and cellular alterations that lead to an anatomic and functional impairment of the neurotransmitter systems. As formerly mentioned, events that lead to the unnatural increase of protein deposits that comprise extracellular amyloid plaques, NFTs, and the further decline of synaptic connections in selective brain areas are conditions evidenced in AD [89]. The product obtained by the proteolytic cleavage of the amyloid precursor protein (APP) by β- and γ-secretases is represented by the Aβ. The Aβ resulted by this proteolytic cleavage is an element of amyloid plaques. The Aβ may be observed in various forms such as in soluble, aggregated, oligomeric, protofibrillar, and fibrillary [90,91]. In this sense, some studies have made clear that oxidative stress is correlated with its oligomeric form being highly toxic [92,93,94]. The initiation of lipid peroxidation processes is related to free radicals connected to Aβ (1–42). These reactive species interact with unsaturated acyl chains of lipid compounds in the lipid bilayer [95,96]. For instance, reactive species as 4-hydroxy-2-nonenal (HNE) and acrolein are generated by the lipid peroxidation process [14]. Such compounds through an interaction with proteins and enzymes may intensify the activity of free radicals processes induced by Aβ (1–42) [96,97]. In cells, in physiological conditions, endogenous antioxidant agents and enzymes counteract damage and oxidative stress. Nonetheless, high levels of redox metal ions, unsaturated lipids, oxygen, and antioxidant systems that are not entirely efficient expose the brain more to oxidative stress. A reduction in antioxidant enzymes levels has been detected in AD and in the mild cognitive impairment (MCI) brain, which makes them exposed to toxic effects induced by Aβ (1–42) [98]. In AD, another aspect of the effect determined by oxidative damage is characterized by elevated levels of protein, lipid, DNA, and RNA oxidation as well as dysfunctions in neurons and death [99,100]. In light of this, therapeutic approaches are aimed at boosting cerebral defenses and ameliorating antioxidant defense systems with particular attention on the effects of GSH, its precursors, and glutathione-related enzymes. In AD, NAC has been tested as a medication, being a precursor for GSH production, showing potential effects that represent an alternative as possible future medication [101]. In rodents, through intraperitoneal application, NAC showed a protective effect to counteract acrolein, peroxynitrite, hydroxyl radicals, and oxidative damage caused by 3-nitro-propionic acid (3-NP) and GSH augmentation in the brain and synaptosomes has been observed [69,102,103,104]. An improvement in learning and memory has been observed by pre-treatment with NAC in mice that have received intracerebroventricular applications of Aβ [105]. Furthermore, among the activities observed, NAC has generated an augmentation in GSH levels, a reduction in ACh levels and ChAT activity, and a protection to counteract the protein and lipid peroxidation induced by Aβ [105]. In an in vivo study using the transgenic APP/PS-1 mice, NAC administered orally in drinking water before the onset of the disease has shown to be able to decrease oxidative damage in neurons, reduce protein and lipid oxidation as well as nitration of proteins and increase the activity of GPx and GSR when compared to control mice [106]. In the AD brain and neuronal cultures exposed to Aβ, cells showed characteristics of apoptosis [107]. The signaling pathways implicated in the apoptosis signaling cascade are modified by a redox status alteration due to NAC [108,109]. Some signaling pathways related to the apoptosis such as the activation of the Ras/ERK pathway, the stimulation of p35/Cdk5 activity, and the reduction of phosphorylation/deactivation of the MLK3-MKK7-JNK3 signaling cascade are involved in NAC protection against Aβ [108,109,110]. The RAS-ERK pathway is activated by NAC and is preserved from apoptosis [110]. Hence, by the activation of anti-apoptotic signaling pathways, NAC has shown a protective effect against Aβ toxicity. Therefore, NAC may play a part in APP gene transcription through its down-regulation. In fact, APP mRNA levels are not detectable in neuroblastoma cells. Such activity can be connected to a diminished binding activity of NF-κB, which is increased by oxidative damage and Aβ [111]. An important neuro-inflammatory component is also observed in AD. Microglia and neurons receive GSH by astrocytes that represent their major provider. Toxic inflammatory mediators and free radicals released by astrocytes during chronic inflammation and oxidative damage accelerate the activation of microglia and neurodegeneration [112]. In human microglia and astrocytes, the release of inflammatory cytokines and the activation of the inflammatory pathway has been associated with a decrease of the amounts of intracellular GSH [113]. Furthermore, NAC promotes both the inhibition of the NF-κB factor and the inducible nitric oxide synthase (iNOS) enzyme with a consequent decrease of the production of NO and inflammatory cytokines [114]. Hence, a protective effect to counteract the neuro-inflammatory element of this pathology may be offered by the increase of the concentration of GSH through NAC in glial cells and astrocytes.

## 6. NAC in Neuropathic Pain

A further relevant field that should be taken into consideration in order to be more investigated is represented by the possible potential applications of NAC in neuropathic pain treatment. This type of chronic pain may be generated by an injury or a disorder that interests the somatosensory nervous system. Clinical incidence is estimated around 7–10% of the general population in which, in 5% of persons, it may be severe [115]. Furthermore, around 40–60% of people may achieve a partial relief, which represents a pain difficult to treat [116]. Treatments may regard the use of some antidepressants such as tricyclic antidepressants and serotonin-norepinephrine reuptake inhibitors, anticonvulsants such as pregabalin and gabapentin, and topical lidocaine [117,118]. The incomplete comprehension regarding the pathophysiological mechanisms of neuropathic pain has encouraged the experimental research to test novel molecules. Nociceptive transmission may be inhibited by NAC, which is related in some measure with its antioxidant activities. Nevertheless, it is not entirely elucidated since NAC may affect the levels of lipid hydroperoxides. Horst et al. have observed that NAC induces a reduction of NO metabolites and an increase in the activity of antioxidant enzymes as GPx and glutathione-S-transferase (GST) in an experimental model of CCI. They have observed that, in CCI rats treated with saline, the ratio between glutathione reduced form (GSH) and glutathione oxidized form (GSSG) was increased while no increase has been observed with the treatment with NAC. The treatment with NAC has increased the activities of GST and GPx and no relevant variations in the GSH/GSSG ratio were observed. However, the treatment with NAC did not influence the levels of H_2_O_2_ but reduce NO metabolites after injury. The anti-hyperalgesic action of NAC seems to not implicate its action as a cysteine precursor for the production of GSH. Hyperalgesia remained at all times in CCI rats but returned to pre-injury values through the treatment with NAC [119]. They have also observed that NAC administration is able to protect against the early increase of lipid hydroperoxide levels in the CCI group by the rise of the ascorbic acid amount and the decrease of total antioxidant capacity at later stages [120]. In a recent study, Horst et al. have demonstrated the role of the P-p38 protein as a mediator in neuropathic pain and nerve regeneration. The treatment with NAC in CCI rats induced a downregulation in the expression of P-p38 and improved recovery of sciatic function [121]. The pain threshold is influenced by the modulation of the Group-II metabotropic glutamate receptors named mGluRs 2 and 3 [122,123,124]. In astrocytes and microglia cells, the endogenous activation of presynaptic mGluRs2/3 is represented by the Sxc-, which is an anti-porter that plays a role in mediating the non-vesicular release of glutamate [125]. System xc- or Sxc- is a membrane antiporter that intervenes in 1:1 exchange of extracellular l-cystine and intracellular l-glutamate, which supports the synthesis of GSH and supplies the intracellular l-cysteine required. Sxc- shows a molecular structure composed by a light chain named xCT and a heavy chain named 4F2hc. The specific transportation of cystine and glutamate through the plasma membrane is mediated by the xCT subunit [126]. Bernabucci et al. have observed the role of NAC both in acute and chronic inflammatory pain. In a model of acute inflammatory pain, the administration of NAC has induced analgesia while this activity was abrogated by pretreatment with sulfasalazine (SSZ) (Scx- inhibitor) and LY341495 (mGlu2/3 receptor antagonist). They have also observed the analgesic effect of NAC in mGlu3-/- mice while no effect in mGlu2-/- mice was detected. In the complete Freund’s adjuvant (CFA) model of chronic inflammatory pain, NAC causes analgesia both after single and repeated administrations while pretreatment with LY341495 abolishes this activity. In a CCI model, NAC loses its analgesic effect after repeated administrations, which induces tolerance. This effect is caused by an ipsilateral reduction in the catalytic subunit of Sxc- levels and bilateral increase expression of an activator of G-protein signaling (AGS3) in the spinal cord. Their data suggest that NAC induced analgesia by enhancing the activity of Sxc- and by reinforcing the endogenous activation of mGluRs2 [127]. Li et al. have observed that NAC attenuates neuropathic pain by matrix metalloproteinase (MMP) inhibition. In vitro and in vivo studies showed that NAC has suppressed the activity of MMP-9 and MMP-2. In a CCI model, NAC blocked the release of IL-1β that represents a critical substrate of MMPs and inhibits the phosphorylation of protein kinase Cg, NMDAR1, and mitogen-activated protein kinases [128]. Sözbir and Nazıroğlu have observed in streptozotocin (STZ)-induced diabetic rats model, a modulatory effect of NAC on dorsal root ganglion (DRG) cells that showed poor levels of GSH. The authors suggest that ROS production induced by diabetes is able to activate the transient receptor potential cation channel (TRPM2) in DRG neurons, which represents the possible cause of the onset of diabetic neuropathic pain. NAC may exert a protective role on oxidative damage and calcium influx by the regulation of the TRPM2 channel showing a protective effect on the gating of such channels. The application of NAC showed a reduction in the lipid peroxidation level of the DRG and brain diabetic rats. NAC exerted protective actions on DRG TRPM2 currents induced by diabetes in brain oxidative damage. The authors suggested that NAC may represent a potential approach in therapies for the prevention of brain damage and neuropathic pain induced by diabetes [129]. Tsai et al. have investigated the action of Sxc- in the onset of knee osteoarthritis (OA) in rats after anterior cruciate ligament transection and medial meniscectomy (ACLT + MMx). They have observed that the intra-articular application of SSZ reduced knee swelling and cartilage destruction in the knees of ACLT + MMx rats and this action was blocked by NAC [130]. Nishio et al. have observed the actions of ROS on synaptic transmission in rat spinal cord substantia gelatinosa (SG) neurons using whole-cell patch-clamp recordings. The authors reported that non-specific ROS scavengers as NAC and phenyl-N-tert-butylnitrone (PBN) significantly suppressed the enhancement of the spontaneous excitatory postsynaptic currents (sEPSCs) in SG neurons, which was induced by tert-butyl hydroperoxide (t-BOOH) [131]. Hacimuftuoglu et al. have observed in a mice model of inflammation induced by intraplantar formalin injection the effect of NAC on tissue injury-induced nociception. To assess the actions of NAC, mice have received an intraperitoneally injection of NAC before intraplantar formalin treatment. Findings revealed that the nociceptive response induced by formalin decreased in the acute phase and even more in the tonic phase [132]. Naik et al. have also observed that intraperitoneal application of NAC showed a decrease of hyperalgesia in CCI-rats [133].

## 7. NAC in Stroke

Stroke represents an event that may cause death and long-term disability. Ischemia-reperfusion causes a series of biochemical damages in cellular and sub-cellular systems. Reperfusion therapy shows a relevant role in the initial treatment of patients that had a stroke, taking in consideration that injury may occur with reperfusion. Potential applications of NAC have been investigated in complications related to neurodegenerative diseases associated with cerebral vascular accidents. Turkmen et al. have observed the effects of NAC and ethyl pyruvate (EP) in a model of ischemic-stroke. In this study, the rats were clamped to the carotid arteries for 1.5 h and to subsequent reperfusion for 2.5 h of reperfusion (I/R group). NAC and EP were administered intraperitoneally after ischemia. A lower percentage of degenerative neurons was observed in rats receiving NAC or EP compared to the I/R group, which indicates that NAC shows neuroprotective efficacy [134]. Sekhon et al. have studied the therapeutic effectiveness of NAC to reduce the ischemia-reperfusion injury in brain tissue generated by a focal cerebral ischemia model in rats. In their study, the focal cerebral ischemia was induced by occluding the middle cerebral artery (MCA) with an intra-luminal suture through the internal carotid artery. Afterward, rats have been observed after reperfusion to assess the score for neurological deficits. After reperfusion, rats were sacrificed and the infarct area in the brain has been evaluated. The authors have noted that NAC-treated rats show a percentage of about 50% reduction in the infarct volume area and a percentage of about 50% reduction in the neurological evaluation score as compared with rats that have not received the treatment. In addition, they observed that the treatment with NAC blocked the expression of TNF-α and iNOS synthase, which was induced by ischemia-reperfusion. Their findings indicate that the previous treatment with NAC reduces cerebral ischemia and reperfusion injury and the protective action observed may be a consequence of the inhibition of TNF-α and iNOS [135]. Liu et al. have evaluated the treatment with NAC and normobaric hyperoxia (NBO) in a rat model of transient focal cerebral ischemia. Their findings displayed that, in post-ischemia, the application of NAC showed a limited action. However, combination treatment causes reductions in tissue infarction and vascular injury. The combined treatments with NAC and NBO displayed a reduction of about 60% of the infarction area as well as a major reduction of the hemispheric swelling volume compared with the treatments with NAC or NBO alone that have shown a lower efficacy. The combination therapy of NAC and NBO has shown a prevention of BBB damage and ameliorated the result of brain lesions [136]. Diabetes may also represent a potential risk factor in stroke, which leads to high levels of methylglyoxal (MG) in blood. Wang et al. have observed in a cerebral ischemia-reperfusion performed in chemical-induced (streptozotocin) and genetic Akita mouse models of Type 1 diabetes that NAC application counteracted the brain injury by restoring GSH formation and normalization of the MG-to-GSH ratio and reducing the diabetic brain infarct area. Their findings may represent a treatment that might help the successful management of stroke in diabetes [137]. Zhang et al., in a transient cerebral ischemia animal model, have observed that the neuroprotective effect of NAC was mediated by hypoxia-inducible factor 1-alpha (HIF-1α) induction and hypoxia-inducible factor 1 (HIF-1) activation. Despite the fact that they provided data to show that heat shock protein 90 (Hsp90) was implicated in the stabilization of HIF-1α by NAC, the exact mechanism by which NAC intervenes in the interaction between Hsp90 and HIF-1α and stabilizes HIF-1α may not be known. They hypothesized that NAC may exert this effect by maintaining redox homeostasis in ischemic brains thanks to its antioxidant properties [138]. Wang et al. have investigated the implications of ROS and δ protein kinase C (δPKC) in neuroprotection caused by remote ischemic post-conditioning (RPostC) in a rat model of focal cerebral ischemia. They have observed that pre-treatment with NAC protected all aspects of RPostC-induced neuroprotection and reversed RPostC-induced inhibition of δPKC activation after reperfusion [139]. Khan et al. have observed the neuroprotective action of NAC in a rat model of stroke in which NAC reduced infarction in coronal sections measured as infarct volume. They observed that the treatment with NAC after onset of ischemia reduced the infarction and improved the neurologic score. The authors have connected this protection with increased GSH amount and reduced apoptotic cell death and the expression of TNF-α, IL-1β and iNOS. The treatment with NAC decreased infarct volume more significantly in cortex and in striatum [140]. Knuckey et al. have observed that NAC partly improved hippocampal neuronal endurance after transient forebrain ischemia. Their findings indicated that free radicals were implicated in the pathogenesis of neuronal death after ischemia but, since the protection is only partial, they hypothesized that free radicals were part of a complex interaction of neurotoxic factors that caused neuronal death after transient forebrain ischemia [141].

## 8. *N*-Acetylcysteine: Clinical Trials in Neurodegenerative Diseases

Over the last several decades, several preclinical studies suggested the potential effectiveness of NAC. In recent times, some clinical trials have been performed to investigate the effects of its administration in order to evaluate strategies in the therapies for the treatment of neurodegenerative diseases (see Table 1). In PD, seven clinical trials have been accomplished in USA (four completed and two ongoing) and one trial is ongoing in France. In AD, three clinical trials have been completed in USA. In neuropathic pain, one clinical trial has been performed in USA. The administration of NAC has been assessed alone or in combination with other compounds. The completed phase 2 clinical trial NCT02212678 included five patients with mild-moderate PD and three healthy controls whereas subjects with a history of asthma or bronchospasm were not included. Four patients with PD and the healthy controls completed the study. The administration of antioxidant supplements prior to the study was not permitted. All participants did not have dementia and were on stable medication treatments for at least one month before enrollment. NAC has been evaluated as oral administration for a treatment of one month with a dose of 6000 mg/day in order to assess changes of GSH levels. The oral administration of NAC showed an increase of cysteine, GSH/GSSG ratio, and catalase while no significant variations in lipid peroxidation, 4-HNE, and malondialdehyde (MDA) were observed. The outcomes of the analysis performed by Magnetic Resonance Spectroscopy (MRS) showed that the oral administration had no significant effect on brain GSH levels. This aspect may be associated with low oral NAC bioavailability and small fractional GSH/GSSG blood responses. Adverse events such as mild indigestion, drooling, and a mild to moderate increase in tremors were observed while a patient showed the freezing of gait that disappeared after treatment suspension. Such events disappeared after the end of the treatment with NAC [142]. The completed phase 1 clinical trial NCT01427517 included three participants with mild to moderate PD, three with Gaucher’s disease, and three healthy controls. The participants did not have dementia. Subjects with a history of asthma or bronchospasm were not considered. The administration of antioxidant supplements was not permitted before enrollment. NAC was administered intravenously through a single infusion of 150 mg/kg and GSH levels in the brain were measured by 7 Tesla MRS. The treatment with NAC has shown an increase in blood GSH redox ratios in patients-treated and healthy controls, which was followed by an increase in brain GSH levels in all nine participants and no adverse events were observed [143]. The completed phase 1/2 clinical trial NCT01470027 included 50 individuals with idiopathic PD and not on any medication (anticholinergic agents permitted). NAC was administered as daily supplementation for one month with two dosages of 1800 mg/day and 36000 mg/day in order to monitor GSH levels increase in the brain when compared to the placebo. After treatment, all participants were assessed by the Unified Parkinson’s Disease Rating Scale (UPDRS), the Mini Mental State Examination (MMSE), the Hamilton Depression Rating Scale (HAM-D) 9-Hole Peg Board Test (9-HPT), the 10-Meter Walk Test, Beck Anxiety Inventory, and the PD Quality of Life Questionnaire (PDQLQ). Changes of GSH levels in the brain were measured by MRS while no serious adverse events were reported. The clinical trial NCT03104725 is currently ongoing. The individuals expected are 41 and the treatment will be performed when they will be hospitalized. This study will include participants with mild PD diagnosed within the past 5 years and under monoamine oxidase (MAO) inhibitor whereas it will exclude individuals under levodopa treatment and already taking antioxidant supplements. NAC will be administered orally to test its ability to inhibit the spontaneous oxidation of central neural DA. The principal outcome measure will be represented by the cerebrospinal fluid (CSF) concentration of cysteine-DA. Further outcomes will regard the levels of other neurochemicals related to catecholamine or of indices of oxidative stress. On the basis of future outcomes, an in-depth study may be performed with an oral dose of NAC of 1000 mg twice per day. The clinical trial NCT02445651 is currently active but not recruiting and the estimated enrollment is of 65 participants. In this study, individuals with PD on stable or on antiparkinsonian medication for at least a month will be included. The primary outcome measures will regard changes in the DA transporter (DAT), which reflects the overall health of the dopaminergic system. The analysis will be performed by Ioflupane (DaTscan) single photon emission computed tomography (SPECT) in order to measure DA function, MRS to measure inflammatory and oxidative stress markers, and neurological measures to assess PD symptoms. Participants will be evaluated by DaTSCAN and MRS before and after treatment. NAC will be expected, respectively, as intravenous doses of 50 mg in 200 mL of dextrose 5% in water (D5W) and oral dose of 1200 mg/day in order to evaluate its potential effect to counteract intracellular damage that leads to DA neurons’ death. Such a clinical trial is the consequence of a preliminary pilot study in which in vitro and in vivo studies have respectively shown that NAC exposure resulted in an increased survival in midbrain dopaminergic (mDA) neurons after exposure to rotenone when compared to no NAC and in an increase of DAT binding in the caudate and putamen in patients that have received NAC treatment [144]. The clinical trial NCT03146130 is also currently ongoing and recruiting in which 70 individuals are estimated to be enrolled. Subjects with mild to moderate impulse control disorder determined by an ECD hyperdopaminergic sub-score under treatment with dopaminergic agonist and/or levodopa and with no change in antiparkinsonian and/or psychotropic treatment in the month before enrollment will be included. The primary outcome will regard the assessment of the variation of the scale of the behavioral evaluation. The administration of NAC will cover a period of 10 weeks and it aims to show an improvement in mild-to-moderate impulse control disorders induced by dopaminergic medications in PD. The primary endpoint will regard the change in score from Part IV of the Ardouin Parkinson’s Behavioral Assessment of Parkinson’s Disease (ECMP) (ECMP IV), which evaluates hyperdopaminergic behaviors. The completed clinical trial NCT02860338 included 900 participants for assessment and treatment of cognitive impairment in several conditions. This study represents an extensive retrospective investigation in which patients were treated with maximal doses of a combination of drugs in which NAC was included. The main outcome measures regarded: objective cognitive testing and functional and behavioral assessments correlated with prescribed standard dementia medications, reductions in benzodiazepine, narcotic, and antipsychotic administration. Among the conditions evaluated, PD e AD were included. This wide analysis may be considered a valuable attempt to assess the comparative effectiveness of dementia drugs and other treatment interventions in cognitively impaired patients. With regards to AD, the completed phase 2 clinical trial NCT01320527 included 135 participants with AD or mild cognitive impairment (MCI). NAC was administered in combination with other compounds in the form of a nutriceutical formulation with a dose of 600 mg/day and the patients were treated for one year. The long-term treatment has demonstrated improvements in cognitive performance and behavioral functions. This trial was based on prior pilot studies aimed to assess the administration of the nutraceutical formulation [145,146]. The completed clinical trial NCT01370954 represents an observational study in which 204 participants with early memory loss were included. Patients were treated with CerefolinNAC^®^, which is a medical food that, among its compounds, include 600 mg of NAC. Such preparation is suggested for the different nutritional requirements of individuals under treatment for early memory loss, mild to moderate cognitive impairment, vascular dementia, or AD. The main outcome measures were aimed to evaluate whether CerefolinNAC^®^ affected the quality of life of participants as measured by the Quality of Life-Alzheimer’s Disease Scale (QOL-AD). The completed phase 2 clinical trial NCT01840345 included 11 participants affected by chronic neuropathic pain. This study was aimed to evaluate the effect of NAC as adjuvant in a combined therapy with opioids. Individuals that showed non-cancerous neuropathic pain, treated with a stable dose of opioids for pain, and used breakthrough pain medications with persistent pain per Visual Analogue Scale (VAS) were included. The oral administration of NAC covered a period of four weeks with a dose of 2400 mg/day. The outcome measures regarded pain intensity measured by using VAS, mood assessed by using the Patient Health Questionnaire (PHQ-9), and stress measured by the Perceived Stress Scale ((PSS). More than 90% of participants treated with NAC have completed the study and four of 10 patients have shown a decrease in average pain ratings even though, with a small group of participants, data must be assessed with caution. No serious adverse events were reported whereas gastroesophageal reflux and nausea were observed respectively in five and four participants. 

Although clinical trials have been performed to demonstrate the relevance of NAC as a valuable compound that may be helpful in neurodegenerative disease therapies, scientific evidence shows that it is necessary to further focus efforts to further investigate the mechanisms of action of this drug.

## 9. Conclusions

NAC represents a compound known for its antioxidant and anti-inflammatory properties. In this review, we highlighted that, thanks to its activities, NAC may be considered as an adjuvant therapy that may be used in combination with conventional therapies in neurodegenerative disorders as PD and AD, neuropathic pain, and stroke. This concept of the association with other drugs is also demonstrated by clinical trials in which NAC has been evaluated in combined therapies. Scientific results encourage future studies for therapeutic treatments due to its effective profile that may be helpful in clinical applications in parallel with other drugs.

## Figures and Tables

**Table 1:** *N*-acetylcysteine: Clinical trials in neurodegenerative diseases (https://clinicaltrials.gov/).

Study Title	Conditions	Status	Treatments	Identifier
Repeated-Dose Oral *N*-acetylcysteine for the Treatment of Parkinson’s Disease	Parkinson’s Disease	Phase 2 completed	Orally (6000 mg/day)	NCT02212678
Intravenous *N*-acetylcysteine for the Treatment of Gaucher’s Disease and Parkinson’s Disease	Parkinson’s Disease	Phase 1 completed	Intravenously (150 mg/kg)	NCT01427517
Does *N*-acetylcysteine Decrease Spontaneous Oxidation of Central Neural Dopamine in Parkinson’s Disease?	Parkinson’s Disease	Recruiting	Orally (4000 mg/day)	NCT03104725
*N*-acetylcysteine for Neuroprotection in Parkinson’s Disease	Parkinson’s Disease	Phase 1/2 completed	Orally (1800 and 3600 mg/day)	NCT01470027
Physiological Effects of Nutritional Support in Patients With Parkinson’s Disease	Parkinson’s Disease	Active, not recruiting	Intravenously/orally (50 mg in 200 mL of D5W and 1200 mg/day)	NCT02445651
Study of the Efficacy of *N*-acetylcysteine (NAC) on Impulse Control Disorders	Parkinson’s Disease	Phase 3 Recruiting	Doses no reported	NCT03146130
Comparative Effectiveness of MCI and Dementia Treatments in a Community-Based Dementia Practice	Parkinson’s and Alzheimer’s Disease	Completed	Doses no reported	NCT02860338
A Clinical Trial of a Vitamin/Nutriceutical Formulation for Alzheimer’s Disease	Alzheimer’s Disease	Phase 2 completed	Orally (600 mg/day)	NCT01320527
NAC-003 P.L.U.S. Program (Progress Through Learning Understanding & Support)	Alzheimer’s Disease	Completed	Orally (600 mg/day)	NCT01370954
The Role of *N*-acetyl-l-cysteine (NAC) as an Adjuvant to Opioid Treatment in Patients With Chronic Neuropathic Pain	Neuropathic Pain	Phase 2 completed	Orally (2400 mg/day)	NCT01840345
